# Role of Hydrocolloids in the Structure, Cooking, and Nutritional Properties of Fiber-Enriched, Fresh Egg Pasta Based on Tiger Nut Flour and Durum Wheat Semolina

**DOI:** 10.3390/foods10102510

**Published:** 2021-10-19

**Authors:** Maria Eugenia Martín-Esparza, Maria Dolores Raigón, Maria Dolores García-Martínez, Ana Albors

**Affiliations:** 1 Food Technology Department, Research Institute of Food Engineering for Development, Universitat Politècnica de València, 46022 Valencia, Spain; analbors@tal.upv.es; 2 Department of Chemistry, Universitat Politècnica de València, 46022 Valencia, Spain; mdraigon@qim.upv.es; 3 Institute for the Preservation and Improvement of Valencian Agro-Diversity, Universitat Politècnica de València, 46022 Valencia, Spain; magarma8@qim.upv.es

**Keywords:** dietary fiber, hydrocolloids, food texture, cooking properties

## Abstract

The aim of this work concerns the manufacturing process of fresh egg tagliatelle labeled as a “source of fiber” based on tiger nut flour and wheat semolina. An attempt to improve the quality attributes and cooking properties of the obtained product was made by means of structuring agents. More specifically, a combination of three hydrocolloids (carboximethylcellulose, CMC; xanthan gum, XG; and locust bean gum, LBG) was tested. A Box–Behnken design with randomized response surface methodology was used to determine a suitable combination of these gums to achieve fewer cooking losses, higher water gain and swelling index values, and better texture characteristics before and after cooking. Positive effects on textural characteristics were observed when incorporating XG into the pasta formulation. Cooking and fiber loss also significantly diminished with the XG-CMC combination over 0.8%. No significant effect was found for the other evaluated parameters. A synergistic interaction between LBG and XG was only significant for the water absorption index. The cooked pasta was considered a source of fiber in all cases.

## 1. Introduction

Edible, sweet, brown-colored tiger nut (*Cyperus esculentus* L.) tubers are widely cultivated in Spain, Burkina Faso, Mali, Niger, and Nigeria [1]. Although it is underutilized in many countries in the world, tiger nut is an important crop in Spain [2], where it is used to produce a milky beverage and has also been employed for animal feed. This tuber is rich in carbohydrates, lipids, fiber, some minerals (potassium, phosphorus, calcium), and vitamins E and C [3]. It is also rich in lipids with a fatty acid profile, similarly to olive and hazelnut oils. The large amount of fiber content (8–15 g/100 g) and omega-6 fatty acids confers this tuber healthy properties [3,4] and plays a key role in the prevention of certain diseases, such as coronary heart disease, colon cancer, diabetes, and obesity [5]. For this reason, several scientific studies on tiger nuts have been conducted. They have focused mainly on the qualitative and quantitative assessments of their nutritional properties and also on the utilization of these components for industrial food purposes. Tiger nut flour (TNF) can be obtained by directly milling clean tubers followed by sieving to achieve the desired homogeneous particle size. This flour has been assessed to produce bakery products [6] and fresh or dry egg pastas [7,8] and for preparing gluten-free (GF) noodles [1] or GF bread with good baking and nutritional characteristics [9,10].

Pasta is a staple food thanks to its simple preparation, variety, versatility, sensory characteristics, and low price [11,12]. Fresh pasta has gained a market share in the last few years. The global fresh pasta market was valued at 1004.6 million USD in 2020 and is expected to grow at a 2.0% CAGR during 2021–2026 [13]. Europe not only purchases the most fresh pasta and consumed 411.49 million tons in 2018 [13] but is also the fastest growing area given its traditional cultural inheritance and good business environment [13]. Adding TNF to the pasta formula is an interesting option for increasing dietary fiber intake, which remains a challenge. Endeavors have been made by several authors to improve nutritional pasta properties, which include pea, oat, teff, quinoa, maize, soy, and amaranth as other plant source flours, mostly to enrich proteins in GF products, dietary fiber, or antioxidants [14,15,16,17,18,19,20,21]. Pasta quality, as affected by an increase in soluble and insoluble fibers, vitamins, and minerals, has been studied by other authors [22,23,24]. The glycemic index can be lowered by including dietary fiber, which additionally may offer other health benefits [22,24,25,26].

Durum wheat proteins can form a continuous viscoelastic network when flour is mixed with water during pasta production. The resulting dough may present optimal properties in mixing and extrusion steps [27], which lead to a final product with better strength and stability. The structure-forming protein in flour (gluten) is also important to reach a correct pasta behavior during cooking, mainly represented by low cooking losses and “al dente” pasta texture. When using GF flours (i.e., tiger nut), lack of gluten must be counteracted by employing ingredients that help to overcome loss of extensibility and elasticity. The literature points out that substances that swell in water (i.e., hydrocolloids), can be utilized to mimic viscoelastic gluten properties by improving acceptability, structural mouthfeel, and shelf life [28]. Hydrocolloids’ film-forming properties can also act as a lubricant in batters and help prevent damage on other formulation ingredients caused by mixing, especially starch granules [29]. The structure of these hydrophilic molecules is variable (linear, branched, with/without chain flexibility) and may interfere with gluten development in relation to their chemical structure. Previously, research has reported that adding hydrocolloids can lead to strong intermolecular hydrogen bonding between the OH groups of gluten proteins and polysaccharide [30,31,32]. Carboxymethylcellulose (CMC), a derivative of cellulose, is a widespread thickening agent employed to modify the viscosity of some food matrices like cake mixes, dairy products, and jellies [16]. Adding CMC (soluble fiber) to cereal-based food has beneficial effects on fasting plasma cholesterol and blood glucose regulation [33]. Non-starch polysaccharides, such as locust bean gum (LBG) and xanthan gum (XG), strongly affect pasta viscoelastic properties. They can be utilized to improve not only its elastic texture but also the mouthfeel and firmness of end products [18]. As far as the authors know, no research is available about evaluating these hydrocolloids being employed to develop fresh egg pasta based on durum wheat semolina (DWS) and TNF.

As reported by [34], response surface methodology (RSM) is a statistical technique that employs quantitative data acquired from suitable experimental designs to establish and simultaneously solve multivariate equations [35]. This tool is effective in optimizing complex processes and has many applications in several food operations [36,37,38,39,40]. By employing tiger nut as a potential source of food nutrients (emphasis is especially placed on the quantity of fiber), the work reported here was done to search the optimum CMC–XG–LBG combination to obtain a high quality “source of fiber”, namely (>3%) tiger nut-based fresh pasta. The most relevant technological pasta properties (consistency, firmness, elasticity, color attributes, cooking loss, water absorption index, swelling index) were assessed. The results may offer a basis for developing fresh tagliatelle using tiger nut-DWS blends with the desired quality and enriched fiber values.

## 2. Materials and Methods

### 2.1. Raw Materials

Commercial TNF and DWS (65% extraction) were respectively supplied by Tigernuts Traders S.L. (L’Eliana, Valencia, Spain) and Harinas Villamayor S.A. (Huesca, Spain). Avícola Llombai S.A. (Llombai, Valencia, Spain) supplied the pasteurized liquid egg (LE). Hydrocolloids (carboxymethylcellulose CMC-3500-4000 cps, locust bean gum LBG-2800 cps, and xanthan gum XG-1400 cps) were, respectively, supplied by Quimica Amtex, S.A. (Mexico City, Mexico), Lbg Sicilia Srl. (Ragusa, Italy) and Shandong Fufeng Fermentation Co., Ltd. (Linyi, China). The same batch formed by the above materials was employed in all the experiments. Raw materials were examined for moisture content, protein, fat, crude fiber, and ash according to AACC methods 44–40.01, 46–10.01, 30–20.01, 32–10.01, and 08–01.01 [41]. The proximate chemical composition of both flours, LE, and hydrocolloids (suppliers sent the data) are summarized in Table 1. 

### 2.2. Experimental Design

The effect of the different factor combinations (three independent variables: CMC, XG, LBG) on the various response variables (randomized RSM) was evaluated by a Box–Behnken design with a quadratic model. The included variables were: nutritional losses during cooking (%P, protein; %F, fat; %M, minerals; %CF, crude fiber; and %DC, digestible carbohydrates), cooking loss (%CL), swelling index (%SI), water absorption index (WAI), CIEL*a*b* color coordinates, chrome (C*_ab_), tone (h*_ab_), firmness (F), consistency (A), and elasticity (S_i_). Both the experimental design and statistical analysis were carried out by version 16.1.17 of the Statgraphics^®^ Centurion XVI statistical software (StatPoint Technologies, Inc., Warrenton, VA, USA, 2011). Both the upper and lower limits of the factor levels were selected after contemplating the preliminary trials (data not provided). Their range went from 0 to 0.8% *w*/*w* (coded as 0 = 0%, 1 = 0.4%, 2 = 0.8%). Fifteen trials were run with three replicates of the central point (Table 2). A multiple regression analysis was followed to assess the significance of the linear, quadratic and interactive effects of factors (CMC, XG, LBG amounts) on the response variables. These parameters were measured in both the uncooked (subscript o) and cooked (subscript c) pasta samples. A second-order polynomial equation describes the regression model (Equation (1)), and every response variable (*Y*) is associated with the obtained linear (*β_i_*), quadratic (*β_ii_*), and interactive (*β_ij_*) regression coefficients, i.e., to the relative weight of every analyzed effect (G_1_-CMC, G_2_-XG, and G_3_-LBG, alone or combined). Constant *β_o_* represents the response if no gum was taken into account.

(1) $$Y=\beta_{o}+\overset{3}{\underset{i=1}{\sum }}\beta_{i}\cdot G_{i}+\overset{3}{\underset{i=1}{\sum }}\beta_{ii}\cdot G_{i}^{2}+\overset{3}{\underset{i=1}{\sum }}\overset{3}{\underset{j>1}{\sum }}\beta_{ij}\cdot G_{i}\cdot G_{j}$$

To better visualize the overall trends, 3-dimensional graphs were employed for the models. Non-significant terms were not included in the model equations to obtain these plots. All the formulations were performed in duplicate.

The basic pasta dough formulation was achieved by mixing tap water (16% *w/w*), DWS (71% *w/w*), and pasteurized LE (13% *w/w*). The quantity of added water was adjusted in earlier tests to obtain dough that was easy to handle and process. TNF was included in recipes at the 42.6% DWS replacement level (*w/w*). This gave a product with a fiber content of about 4%, which was labeled as “source of fiber” (>3 g dietary fiber/100 g food) according to the Nutritional Requirements for Dietary Fiber Foods [42]. The chemical composition of the raw materials was considered to estimate fiber content. 

### 2.3. Pasta Preparation

After weighing (0.001 g accuracy, PFB 300-3, Kern & Sohn GmbH, Balingen), both the liquid (egg/water) and dry (DWS/TNF/gums) ingredients were premixed in an electric cooking device (Thermomix TM-31, Vorwerk Spain M.S.L., S.C., Madrid, Spain) at medium speed, mixing egg and water for 15 s, adding gums to be mixed for 40 s, and then incorporating DWS/TNF powders and kneading for 45 s more yielded a uniform blend. The blends were kneaded in the same cooking device for 2.5 min and then placed inside plastic bags for 20 min for sample relaxation purposes. Then, tagliatelle was made with a pasta-making device (Simplex SP150, Imperia, Italy) coupled to a specific motor (A2500, Imperia, Italy). Dough was laminated by passing it between rollers 5 times before gradually narrowing the gap between rollers to make 1-mm-thick sheets, which were cut into 4-mm-wide tagliatelle. Tagliatelle was left to stand for 10 min to prevent stickiness before cooking began. A temperature of 20 °C was maintained while preparing and analyzing dough. Tagliatelle samples were made to be immediately tested for their mass, dimensions (volume), water content, mechanical properties, and color attributes (see the analysis explained below). There were three replicates (5 for mechanical properties) per pasta formulation. 

### 2.4. Pasta Cooking

The cooking trial for each pasta formulation was done in triplicate. Cooked pasta was prepared by boiling 25 g of 7-cm-long samples in 300 mL of deionized water. Water volume was left at 90% of its initial volume by adding boiling water and covering flasks to prevent loss of evaporation. At 4 min (optimal cooking time for 100% DWS fresh egg tagliatelle according to the AACC method 16–50 [41]), pasta was removed from flasks before quickly stopping the cooking process by adding 50 mL of cold deionized water. Next, pasta samples were drained for 2 min, weighed (0.001 g accuracy, PFB 300-3, Kern & Sohn GmbH, Balingen, Germany), and evaluated for their water absorption index (WI), cooking losses (%CL), swelling index (%SI, volume changes), proximate chemical composition, mechanical properties, and color attributes (analysis explained below).

### 2.5. Proximate Chemical Composition of Both Cooked and Uncooked Pasta Samples

Cooked tagliatelle was analyzed for its water content, crude fiber, protein, ash, and fat according to AACC methods 44–40.01, 46–10.01, 30–20.01, 32–10.01, and 08–01.01 [41]. Digestible carbohydrates were calculated by difference. There were three replicates per formulation. Moisture content was immediately analyzed after cooking; for the other chemical measurements, cooked pasta was freeze-dried (Telstar, Lyoalfa-6, Azbil, Spain) for 24 h at 0.1 mbar and stored at room temperature in sealed polyethylene bags until further analyses. The proximate chemical composition of the raw materials was employed to calculate that of the uncooked pasta samples to know the corresponding percentage losses caused by cooking. 

### 2.6. Pasta Technological Properties

The water absorption index (WAI, g/g) was calculated from both mass gain and increased water content after cooking. Cooking loss (quantity of solid substance lost to cooking water; %CL) was determined by the AACC-approved method 16–50 [41], with some modifications. After cooking, both the cooking and rinse waters were collected and left in an aluminum container to be evaporated to dryness by two steps: placing in an air oven at 100 °C to reduce 2/3 volume and freeze-drying (Telstar, Lyoalfa-6, Azbil, Spain). The residue was weighed and indicated as a percentage of starting material. There were three replicates per formulation. Tagliatelle dimensions (thickness, length, width) were taken using a caliper (PCE-DCP 200N, PCE Ibérica S.L., Albacete, Spain).

Pasta swelling (%SI) was expressed as the relative volume changes between the cooked and uncooked samples. There were three replicates per formulation.

Tagliatelle color measurements were taken over the surface reflectance spectra obtained by a spectrocolorimeter (Minolta CM-3600D) from 400 to 700 nm (iluminant D65, 10° standard observer) on a white background. Determinations were made for all the pasta formulations in triplicate both before and after cooking (0 and 4 min). The CIEL*a*b* color coordinates L* (lightness), a* (redness-greenness), and b* (yellowness-blueness) were obtained from the reflectance spectra, and the results were expressed in terms of chromatic magnitudes: color saturation ($C_{ab}^{*}=\sqrt{a^{*2}+b^{*2}}$) and hue angle ($h_{ab}^{*}=arctg\frac{b^{*}}{a^{*}}$). 

A Texture Analyzer (TA.XT2, Stable Micro Systems, Godalming, UK), coupled to a PC with data acquisition and version 1.22 of the Texture Exponent software (Stable Micro Systems), was employed to determine the mechanical properties. Tests were run in accordance with the AACC Method 16–50 [41]. Five 7-cm-long adjacent strands were cut using the A/LKB-F cutting probe at 0.17 mm/s until total sample deformation was achieved. A 5-kg load cell was employed. At least five replicates for the uncooked and cooked pasta were obtained and also for all the pasta formulations. Cooked samples were analyzed just after the cooking procedure, as described in Section 2.4. To evaluate changes in pasta texture while cooking, three parameters were taken into account: (i) force needed to cut tagliatelle (F) as a measure of firmness; (ii) the area compressed under the force-time curve (A) from the initial test time to the maximum cut force, which represents dough consistency. (iii) The initial slope of the force-time curve (S_i_), which is related to the elasticity modulus, offers an idea of products’ solid nature. 

### 2.7. Statistical Analysis

Version 16.1.17 of the Statgraphics^®^ Centurion XVI.I statistical software (StatPoint Technologies, Inc., 2011) was employed to fit the multiple regression models to the experimental data. This enabled the linear, quadratic, and interactive effects of hydrocolloids CMC, XG, and LBG on the selected dependent variables to be evaluated (*p* < 0.05). This statistical software was also used to produce surface response plots.

## 3. Results and Discussion

The experimental values for the cooking, optical and mechanical properties, and the chemical changes due to cooking for each experimental run are presented in Table 2. The results of the 15 runs were fitted to a second-order polynomial equation (Equation (1)). The removal of the non-significant terms (*p* < 0.05) was considered (stepwise regression).

The fitted model’s goodness was assessed by an analysis of variance (ANOVA; Table 3), based mostly on probability (*p*-value) and the Fisher variation test (F-value), to gain a measurement of how much variability in the observed response values can be explained by the experimental factors and their interactions [43]. A *p*-value less than 0.0500 indicates that the model is statistically significant; therefore, only models where this value was greater than 0.0500 are shown in Table 3. 

A Student’s *t*-test was run to analyze the significance of the parameters’ regression coefficients. Table 4 provides the results obtained for the *t*-values, the corresponding *p*-values, and the parameter estimates. 

The model’s insights can be also obtained from determination coefficients (see Table 5 and Table 6). R^2^ quantitatively evaluates the correlation between the experimental data and the predicted responses, while R^2^_adj_ defines the satisfactory fit of the polynomial model to experimental data. In practice, a model can be considered fairly good for describing the influence of the variable(s) when the coefficient of determination (R^2^) is at least 80% [43] or the R^2^_adj_ values exceed 70% [44].

### 3.1. Mechanical Properties of the Uncooked and the Cooked Fresh Egg Pasta

The instrumental parameters of elasticity, firmness, and stickiness may be associated with consumer pasta acceptability. The expected high-quality cooked pasta should display good texture, resist stickiness and surface disintegration, and have a firm but consistent and elastic structure (“al dente”). Table 5 summarizes the estimated regression coefficients (Y_Fo_, Y_Fc_, Y_Sic_, Y_Ac_) of the second-order model, which were obtained for the mechanical properties of the uncooked/cooked tagliatelle and include the fitted parameters from the ANOVA. The predictive models developed for not only the firmness of the uncooked (F_o_) and cooked (F_c_) pasta but also for cooked pasta consistency (A_c_) and elasticity (S_ic_) were deemed suitable because the model significance and the R^2^_adj_ values levels were satisfactory. The lack-of-fit parameter was always non-significant (*p* > 0.05), and the Durbin–Watson statistic *p*-value exceeded 0.05, which meant no indication of serial autocorrelation in the residuals at the 5% significance level.

**Figure 1:**
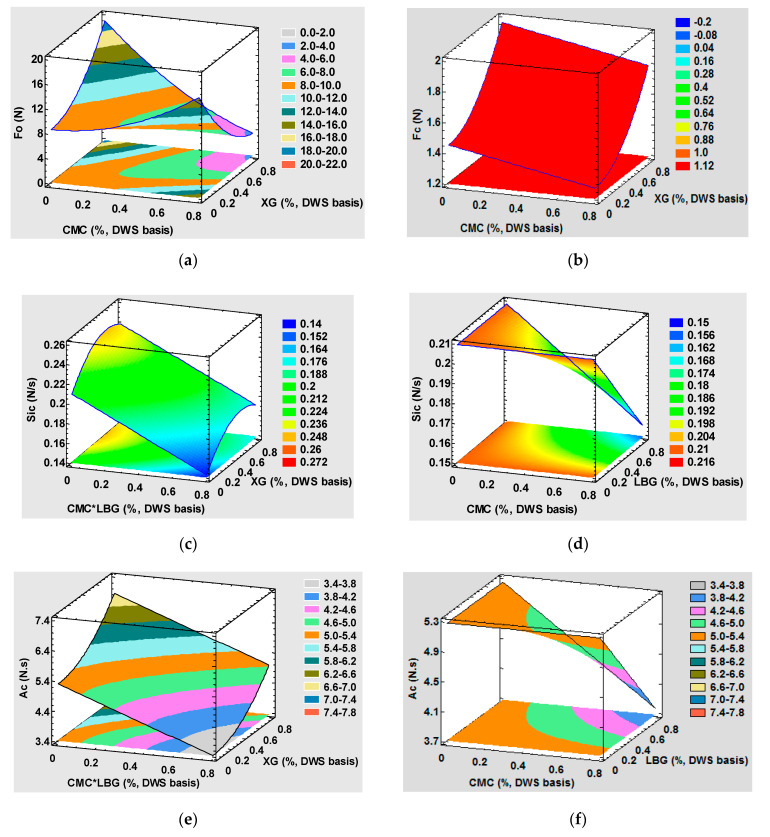
(**a**–**f**). Effect of the CMC, GX, and LBG levels on the uncooked and cooked firmness (F_o_ and F_c_, respectively) and on cooked elasticity (S_ic_) and consistency (A_c_). CMC, carboximethylcelullose; XG, xanthan gum; LBG, locust bean gum.

Figure 1 depicts the response surface plots for the various mechanical parameters of the uncooked (a) and cooked pasta (b–f). The β and *p* values in Table 4 and Table 5 show that the presence of XG significantly and positively influenced the mechanical fresh pasta properties at the tested concentration range. The firmness of the uncooked and cooked pasta quadratically rose with XG concentration. This impact was much stronger before cooking. Figure 1a,b show that the addition of 0.8% XG to the tiger-nut-based pasta brought about increases in the firmness of the uncooked (F_o_) and cooked (F_c_) tagliatelle pieces of 125.52% and 36.31% (values calculated from models), respectively. XG has been reported to enhance the firmness of bran-enriched spaghetti [45], composite semolina-flaxseed spaghetti [46], and GF tiger nut noodles [1]. Cooked pasta elasticity (Figure 1c), particularly consistency (Figure 1e), also improved (a maximum rise of 30.87% at the XG 0.8% concentration for consistency and one of 17.66% at the XG 0.56% concentration for elasticity). These results show the possibility of obtaining a better structure with continuous protein matrix entrapping starch granules, which absorb water and gelatinize with no major losses due to cooking. Soluble gums, such as CMC, LBG, and XG, have the potential to affect the internal pasta structure because of their interaction with starch and protein. The authors of ref [18] put forward the notion that forming a network by soluble fiber around starch granules could result in better cohesiveness in a pasta structure between protein and starch. In [5], the authors report that the rheological behavior of a tiger nut–wheat semolina composite dough was impacted by this XG at a 1% concentration because a more cohesive structure was obtained. Therefore, adding XG to formulations helps to enhance dough resistance to deformation. After hydrating this hydrocolloid, it can fill up any free space in the system, which makes dough structure stronger. As a previous study reports [47], adding up to 1% XG to corn-bean pasta results in a more compact internal structure with visible starch granule agglomerates embedded in the fibrous protein-gum matrix. In line with these results, it would be interesting to employ a XG concentration of approximately 0.6% to achieve improved textural characteristics to approach the sought “al dente” point.

Uncooked pasta firmness also improved (up to 47.32%) when only CMC was employed and had a quadratic positive effect (β value in Table 5; Figure 1c). This parameter slightly decreased after cooking (linear negative effect; β value in Table 5) after adding carboxymethylcellulose (with 12.48% at the 0.8% concentration). A significant and negative CMC and XG interaction was noted (Table 5; Figure 1a). Thus, uncooked pasta firmness significantly diminished when both hydrocolloids were combined. LBG affected only cooked pasta’s consistency and elasticity when combined with CMC (Table 5; Figure 1d,f) but did not affect the mechanical response when employed alone.

We can conclude from these results that only XG implies a better, ready-to-eat tagliatelle texture within the test range, and the combination of CMC and LBG or XG ought to be avoided. No synergistic effect was observed between LBG and XG.

### 3.2. Cooking Quality and Color Attributes of Uncooked and Cooked Fresh Egg Pasta

Table 6 presents the regression summary and ANOVA for cooking quality (WAI, %CL, and %SI) and fiber loss during cooking (%FL). At the 95% confidence level, %CL and %FL proved to be significant variables for model construction (R^2^_adj_ > 70%). The model was less suitable for the swelling index (%SI, R^2^_adj_ = 62.75%) and the water absorption index (WAI, R^2^_adj_ = 61.56%). However, an explanatory data analysis was run, which gave a reasonable initial solution for describing the tendency of these parameters.

**Figure 2:**
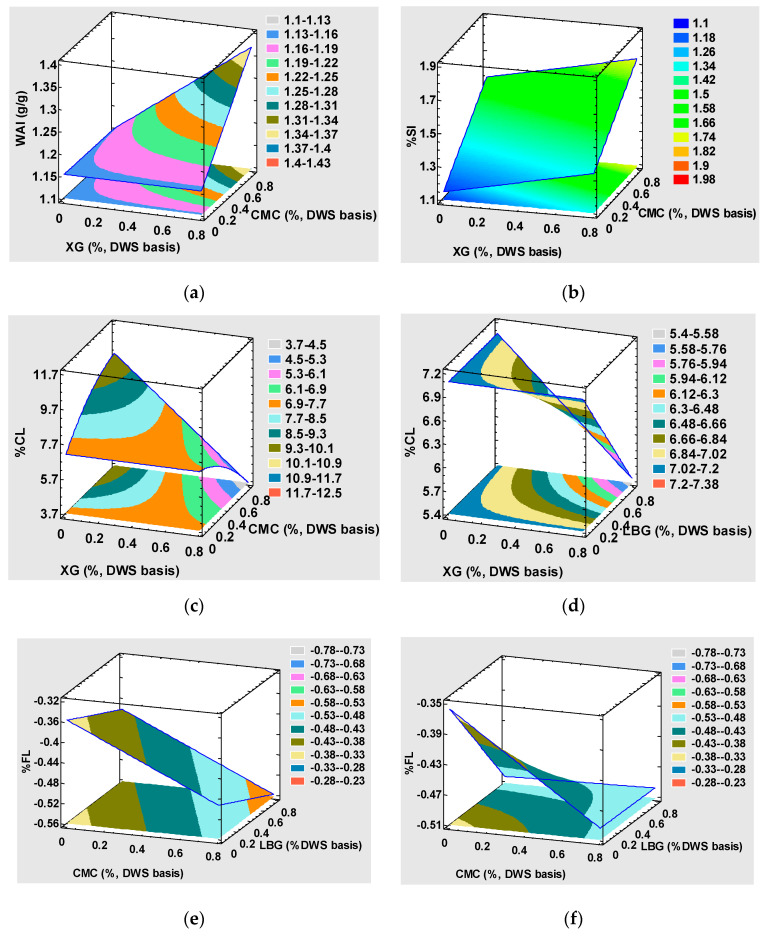
(**a**–**f**). Effect of the CMC, GX, and LBG levels on water absorption index (WAI), the swelling index (%SI), cooking loss (%CL), and fiber loss (%FL). CMC, carboximethylcelullose; XG, xanthan gum; LBG, locust bean gum.

The model developed for %SI and WAI was less predictive with an R^2^_adj_ of 61.56% and 62.75%, respectively. This can be partly explained by the experimental response variables’ narrow range (0.1–1.39 for WAI; 0.94–1.69 for %SI). Regression coefficients (Table 6) and surface plots (Figure 2a,b) were generated for the use models, as they give a reasonable initial solution for describing the quality response of both %SI and WAI. CMC (β = 0.510) and XG (β = 0.238) displayed a linear positive effect on swelling capacity, and a significant synergetic effect on WAI of both hydrocolloids was found (Figure 2a). This tendency is supported by greater swelling possibly being related to a large quantity of water bonded to proteins and starch because WAI displayed a similar pattern. CMC did not affect WAI, like other authors have found when substituting 0.25–1.5% wheat semolina for this hydrocolloid [22]. Cooking loss (%CL) is a pasta-quality measure that expresses resistance to disintegration when boiling. Figure 2c and 2d depict a drastic drop in %CL when combining XG and CMC (β = −10.003) or LBG (β = −2.542) in the pasta formula and, respectively, resulted in less cooking loss of 47.35% or 22.97% when these gums were employed at the 0.8% concentration. The obtained results also revealed that CMC increased the cooking loss by 43.03% when used at 0.8% concentration. However, it was not affected when XG or LBG was employed alone within the test range. From this viewpoint, the XG and CMC combination at 0.8% is recommendable. For wheat-based pasta, %CL is dependent on the degree of starch gelatinization and the strength of the retrograded starch network that surrounds gelatinized starch [48]. Solid loss while cooking is due mainly to the solubilization of loosely bound gelatinized starch from the product’s surface [48]. In non-conventional pasta, starch polymers are entrapped less efficaciously in the matrix, which confers products a high CL, as expected given the concordance between the lower cooking loss and the better mechanical response obtained when XG was added. A significant difference in digestible carbohydrate losses while cooking is also expected. However, fiber loss during cooking was the only significant chemical component for model construction (of those assessed) (Table 6, Figure 2e,f). The ready-to-eat product had a final fiber content that went from 3.1 (0.6) for trial 13 to 3.6 (0.5) for trial 15. These results allow it to be labeled as a “source of fiber” (>3 g dietary fiber/100 g food) in line with Nutritional Requirements for Dietary Fiber Foods [42].

The color parameters were not statistically related (*p* < 0.05) to the hydrocolloids used within the test range.

## 4. Conclusions

The results from this manuscript address the improved nutritional value and fair techno-functional properties obtained with fresh tiger nut-based tagliatelle when XG was employed as a structural agent. Marked fiber and fat enhancement (rich in oleic and linoleic acids) contents, along with mineral enrichment, may be attained in tiger nut pasta. XG at 0.8% concentration considerably improved the textural characteristics and, accordingly, fresh pasta’s cooking behavior. This means that a better structure with a continuous protein matrix to entrap starch granules is feasible. 

It was not possible to accomplish an adequate hydrocolloid combination (CMC, XG, and LBG) within the test range (0–0.8%) with the RSM analysis. Nonetheless, the obtained results showed that employing XG at a concentration of about 0.6% would be interesting for obtaining better ready-to-eat, fresh pasta textural characteristics. Combining this gum with CMC at 0.8% can considerably reduce cooking losses while cooking. The cooked pasta can be labeled as a “source of fiber” (>3 g dietary fiber/100 g food) in all cases.

## Figures and Tables

**Table 1:** Proximate chemical composition of: tiger nut flour (TNF); durum wheat semolina (DWS); liquid egg (LE) (g/100 g); hydrocolloids (CMC, XG, LBG). The mean values of three replicates are provided (standard deviation) for TNF, DWS, and LE.

	DWS	TNF	LE	CMC *	XG *	LBG *
Water	13.67 (0.03)	8.83 (0.05)	79 (2)	10	15	12
Protein	13.2 (0.7)	4.95 (0.07)	11.4 (0.6)	-	-	-
Fat	0.90 (0.05)	25.07 (0.02)	7.83 (0.98)	-	-	-
Ash	1.71 (0.07)	2.05 (0.04)	0.60 (0.04)	-	13	1
Dietary Fiber	10.00 (0.02)	15.85 (0.03)	-	90	72	87
DC **	60.54 (0.02)	43.25 (0.03)	0.91 (0.02)	-	-	-

* Suppliers provided the data. ** Digestible carbohydrates were calculated by the difference.

**Table 2:** Response variable values for the various pasta formulations that correspond to the experimental design in accordance to the levels of the three gum concentrations (CMC, carboxymethylcellulose; LBG, locust bean gum; XG, xanthan gum).

	Factors *			Response Variable **																						
Run Order	CMC	XG	LBG	Y_Fo_	Y_Sio_	Y_Ao_	Y_Fc_	Y_Sic_	Y_Ac_	Y_WI_	Y_%CL_	Y_%SI_	Y_L*o_	Y_a*o_	Y_b*o_	Y_C*ab,o_	Y_h*ab,o_	Y_L*c_	Y_a*c_	Y_b*c_	Y_C*ab,c_	Y_h*ab,c_	%P	%L	%A	%FL
1	+1	+1	0	6.15	0.48	18.38	1.72	0.21	5.89	1.39	2.424	1.69	55.89	4.82	19.37	19.96	1.33	62.00	2.06	13.67	13.83	1.42	−0.13	−0.16	−0.15	−0.54
2	−1	+1	0	19.59	1.41	30.64	1.98	0.24	6.75	1.10	6.64	1.28	55.69	5.09	18.58	19.26	1.30	62.92	2.03	14.07	14.22	1.43	−0.13	−0.06	−0.22	−0.50
3	0	−1	+1	14.01	1.13	21.69	1.23	0.18	4.44	1.18	8.11	1.36	56.09	5.32	20.56	21.24	1.32	61.42	2.55	14.94	15.16	1.40	−0.09	−0.002	−0.15	−0.51
4	+1	−1	0	14.89	1.34	22.34	1.27	0.19	4.99	1.17	−	1.68	56.25	4.93	20.17	20.76	1.33	56.72	1.95	12.08	12.24	1.41	−0.10	0.03	−0.08	−0.49
5	0	0	0	5.29	0.36	10.68	1.47	0.24	5.03	1.26	6.84	1.49	55.36	4.79	20.21	20.77	1.34	61.18	2.23	13.71	13.89	1.41	−0.11	−0.04	−0.14	−0.46
6	0	+1	+1	7.62	0.57	22.68	1.87	0.21	6.26	1.34	5.61	1.69	56.23	4.67	19.40	19.96	1.33	62.86	1.50	12.75	12.84	1.45	−0.10	−0.08	−0.17	−0.54
7	0	0	0	5.30	0.09	10.76	1.47	0.22	4.75	1.23	7.08	1.52	56.43	4.94	20.86	21.43	1.34	60.71	2.39	14.52	14.72	1.41	−0.06	−0.19	−0.18	−0.50
8	−1	0	−1	12.87	0.63	15.59	1.62	0.27	5.77	1.20	7.67	1.37	56.61	5.26	19.73	20.42	1.31	61.55	2.16	13.22	13.39	1.41	−0.16	−0.12	−0.18	−0.39
9	−1	−1	0	9.07	0.34	11.40	1.50	0.20	5.71	1.15	6.95	0.94	54.63	4.87	20.25	20.83	1.34	61.17	2.47	14.44	14.65	1.40	−0.11	−0.06	−0.18	−0.40
10	0	+1	−1	9.89	1.35	21.26	1.92	0.25	7.32	1.16	6.25	1.39	58.24	4.37	18.39	18.90	1.34	62.98	2.12	13.86	14.03	1.42	−0.10	−0.14	−0.13	−0.51
11	+1	0	−1	7.78	0.87	18.05	1.57	0.22	6.20	1.31	7.12	1.59	59.15	3.91	17.81	18.23	1.36	61.69	2.35	14.28	14.48	1.41	−0.13	0.07	−0.04	−0.53
12	0	−1	−1	11.60	0.67	11.84	1.28	0.21	4.77	1.15	−	1.30	56.65	5.13	20.29	20.93	1.32	64.55	1.97	14.24	14.38	1.43	−0.09	−0.13	−0.24	−0.43
13	0	0	+1	6.75	0.59	17.71	1.54	0.22	5.25	1.14	6.60	1.48	53.94	5.69	20.97	21.73	1.31	60.38	2.05	13.37	13.53	1.42	−0.10	−0.15	−0.06	−0.57
14	+1	0	+1	6.34	0.45	12.68	1.39	0.19	4.27	1.22	5.60	1.52	56.06	4.91	20.39	20.97	1.33	60.73	1.96	13.53	13.68	1.43	−0.12	−0.17	0.0005	−0.53
15	0	0	0	5.67	0.40	11.82	1.49	0.24	4.71	1.18	7.23	1.57	53.84	5.55	21.26	21.97	1.32	60.96	2.08	13.77	13.92	1.42	−0.11	−0.09	−0.13	−0.52

* Factors CMC, XG, and LBG stand for carboxymethylcellulose; xanthan gum; locust bean gum; −1 = 0% *w/w*; 0 = 0.4% *w/w*; +1 = 0.8% *w/w*;.** Response variables Y_Fo_, Y_Fc_, Y_Sio_, Y_Sic_, Y_Ao_, Y_Ac_, Y_WI_, Y_%CL_, Y_%SI_,Y_L*o_, Y_a*o_, Y_b*o_, Y_C*ab,o_, Y_h*ab, o_,Y_L*c_, Y_a*c_, Y_b*c_, Y_C*ab,c_, Y_h*ab,c_ stand for elasticity (S_i_), firmness (F), consistency (A), water absorption index (WI), cooking loss (%CL), swelling index (%SI), lightness (L*), redness (color coordinate a*), yellowness (color coordinate b*), chrome (C*_ab_), hue angle (h*_ab_), losses of proteins (%P), lipids (%L), minerals (%A), and fiber (%FL), respectively. Subscripts *o* and *c* refer to the uncooked and cooked pasta samples, respectively.

**Table 3:** Analysis of variance of response surface models.

Variables	Sources Of Variations	SS	DF	MS	F-Value	*p*-Value
	Model	211.398	3	70.466	18.85	0.0001
F_o_ (N)	Residual	41.131	11	3.739		
	Corrected total	252.529	14			
	Model	0.687	2	0.344	61.58	<0.0001
F_c_ (N)	Residual	0.067	12	0.006		
	Corrected total	0.754	14			
	Model	0.006	3	0.002	14.63	0.0004
S_ic_ (N/s)	Residual	0.002	11	0.0001		
	Corrected total	0.008	14			
	Model	9.012	2	4.506	26.12	<0.0000
A_c_ (N·s)	Residual	2.071	12	0.173		
	Corrected total	11.083	14			
	Model	0.057	1	0.057	20.82	0.0005
WAI (g/g)	Residual	0.036	13	0.003		
	Corrected total	0.092	14			
	Model	22.383	4	5.596	31.57	0.0001
%CL	Residual	1.418	8	0.177		
	Corrected total	23.802	12			
%SI	Model	0.361	2	0.180	12.79	0.0011
	Residual	0.169	12	0.014		
	Corrected total	0.530	14			
%FL	Model	0.031	4	0.008	15.14	0.0003
	Residual	0.005	10	0.001		
	Corrected total	0.037	14			

SS, sum of squares; DF, degree of freedom; MS, mean square; F-value, Fisher test; *p*-value, probability.

**Table 4:** Regression results from the data.

Variables		Coefficient Estimate	Standard Error	95% Confidence Interval Low	95% Confidence Interval High	*t*−Value	*p*−Value
F_o_ (N)	β_o_	8.561	0.93	6.52	10.60	9.25	<0.0001
	CMC*CMC	12.015	3.04	5.33	18.70	3.95	0.0023
	XG*XG	16.790	3.01	10.17	23.41	5.58	0.0002
	CMC*XG	−36.539	5.03	−47.622	−25.46	−7.26	<0.0001
F_c_ (N)	β_o_	1.449	0.04	1.36	1.54	35.19	<0.0001
	CMC	−0.226	0.07	−0.38	−0.07	−3.19	0.0078
	XG*XG	0.822	0.08	0.65	0.99	10.51	<0.0001
S_ic_ (N/s)	β_o_	0.209	0.01	0.19	0.22	32.13	<0.0001
	XG	0.132	0.03	0.06	0.20	4.04	0.0019
	XG*XG	−0.118	0.04	−0.20	−0.03	−3.06	0.0109
	CMC*LBG	−0.084	0.02	−0.12	−0.05	−5.01	0.0004
A_c_ (N·s)	β_o_	5.275	0.187	4.868	5.683	28.19	<0.0001
	XG*XG	2.544	0.435	1.597	3.491	5.85	0.0001
	CMC*LBG	−2.340	0.585	−3.614	−1.065	−4.00	0.0018
WAI (g/g)	β_o_	1.154	0.019	1.113	1.194	61.97	<0.0001
	CMC*XG	0.343	0.075	0.180	0.505	4.56	0.0005
%CL	β_o_	7.084	0.243	6.523	7.645	29.142	<0.0001
	CMC	6.614	1.435	3.306	9.923	4.611	0.0017
	CMC*CMC	−3.505	1.492	−6.945	−0.065	−2.350	0.0467
	CMC*XG	−10.003	1.135	−12.620	−7.387	−8.817	0.0000
	CMC*LBG	−2.542	0.789	−4.361	−0.723	−3.222	0.0122
%SI	β_o_	1.144	0.071	0.990	1.299	16.162	<0.0001
	CMC	0.501	0.113	0.264	0.756	4.524	0.0007
	XG	0.238	0.105	0.009	0.466	2.263	0.0429
%FL	β_o_	−0.358	0.020	−0.402	−0.314	−18.089	<0.0001
	CMC	−0.171	0.036	−0.252	−0.090	−4.694	0.0008
	XG	−0.076	0.020	−0.121	−0.031	−3.760	0.0037
	LBG	−0.179	0.045	−0.280	−0.078	−3.956	0.0027
	CMC*LBG	0.221	0.088	0.024	0.418	2.504	0.0312

**Table 5:** Constant values (β_o_) and significant coefficients (β) at the 95% confidence interval of the stepwise multiple regression model for mechanical properties firmness (F), elasticity (Si), and consistency (A).

		F_o_ (N)	F_c_ (N)	S_ic_ (N/s)	A_c_ (N·s)
	Constant (β_o_)	8.561	1.449	0.209	5.275
β	CMC	*ns*	−0.226	*ns*	*ns*
	XG	*ns*	*ns*	0.132	*ns*
	LBG	*ns*	*ns*	*ns*	*ns*
	CMC*CMC	12.015	*ns*	*ns*	*ns*
	XG*XG	16.790	0.822	−0.118	2.544
	LBG*LBG	*ns*	*ns*	*ns*	*ns*
	CMC*XG	−36.539	*ns*	*ns*	*ns*
	CMC*LBG	*ns*	*ns*	−0.084	−2.340
	XG*LBG	*ns*	*ns*	*ns*	*ns*
	Lack of fit	41.037	0.06682	0.001716	2.0096
	Pure error	0.094	0.00018	0.000284	0.0614
	Lack of fit *p*-value	0.999	0.999	0.823	0.997
	R^2^	83.71	91.121	79.97	81.317
	R^2^adj	79.27	89.641	74.50	78.204
	Standard error of est.	1.93	0.075	0.012	0.415
	Mean absolute error	1.40	0.048	0.007	0.324
	Durbin–Watson statistic (*p*-value)	1.417 (0.126)	2.214 (0.637)	2.727 (0.919)	2.047 (0.485)

Independent variables: CMC (carboximethylcelullose); XG (xanthan gum); LBG (locust bean gum). Subscripts o and c refer to the uncooked and cooked pasta samples, respectively. Only the significant relations are shown. Analysis of variance at the 95% confidence level. *ns*, no significant effect at level < 5%.

**Table 6:** Constant values (Y_0_) and significant coefficients (β) at 95% confidence interval of the stepwise multiple regression for cooking properties water absorption index (WAI), cooking loss (%CL), and swelling index (%SI).

		WAI (g/g)	%CL	%SI	%FL
	Constant (β_o_)	1.154	7.084	1.144	−0.358
β	CMC	*ns*	6.614	0.510	−0.171
	XG	*ns*	*ns*	0.238	−0.076
	LBG	*ns*	*ns*	*ns*	−0.179
	CMC*CMC	*ns*	−3.505	*ns*	*ns*
	XG*XG	*ns*	*ns*	*ns*	*ns*
	LBG*LBG	*ns*	*ns*	*ns*	*ns*
	CMC*XG	0.343	−10.003	*ns*	*ns*
	CMC*LBG	*ns*	*ns*	*ns*	0.221
	XG*LBG	*ns*	−2.542	*ns*	*ns*
	Lack of fit	0.032733	1.3395	0.165749	0.003120
	Pure error	0.003267	0.0785	0.003369	0.002074
	Lack of fit *p*-value	0.244	0.135	0.096	0.870
	R^2^	61.56	94.04	68.07	85.83
	R^2^adj	58.61	91.06	62.75	80.16
	Standard error of est.	0.052	0.421	0.119	0.023
	Mean absolute error	0.040	0.289	0.09	0.014
	Durbin−Watson statistic (*p*-value)	1.676 (0.250)	1.737 (0.285)	1.552 (0.185)	1.553 (0.186)

Independent variables: CMC (carboximethylcelullose); XG (xanthan gum); LBG (locust bean gum). Subscripts o and c refer to the uncooked and cooked pasta samples, respectively. Only significant relations are shown. Analysis of variance at the 95% confidence level. *ns*, no significant effect at level < 5%.
